# Rosmarinic Acid Attenuates Airway Inflammation and Hyperresponsiveness in a Murine Model of Asthma

**DOI:** 10.3390/molecules21060769

**Published:** 2016-06-13

**Authors:** Zhengmin Liang, Yangfeng Xu, Xuemei Wen, Haiying Nie, Tingjun Hu, Xiaofeng Yang, Xiao Chu, Jian Yang, Xuming Deng, Jiakang He

**Affiliations:** 1 College of Animal Science and Technology, Guangxi University, Nanning 530005, China; liangzhengmin815@163.com (Z.L.); yangfengxu1992@gmail.com (Y.X.); xuemeiwen1992@163.com (X.W.); 15296494675@163.com (H.N.); tingjunhu@126.com (T.H.); yangjian0104@163.com (J.Y.); 2 College of Animal Science and Veterinary Medicine, Jilin University, Changchun 130062, Jilin, China; xiaofengyang@hotmail.com (X.Y.); 421591993@163.com (X.C.); Dengxm@jlu.edu.cn (X.D.)

**Keywords:** RA, preventive, asthma, MAPK, NF-κB, mRNA expression

## Abstract

Rosmarinic acid (RA) has numerous pharmacologic effects, including anti-oxidant, anti-inflammatory, and analgesic effects. This study aimed to evaluate the preventive activity of RA in a murine model of asthma and to investigate its possible molecular mechanisms. Female BALB/c mice sensitized and challenged with ovalbumin (Ova) were pretreated with RA (5, 10 or 20 mg/kg) at 1 h before Ova challenge. The results demonstrated that RA markedly inhibited increases in inflammatory cells and Th2 cytokines in the bronchoalveolar lavage fluid (BALF), significantly reduced the total IgE and Ova-specific IgE concentrations, and greatly ameliorated airway hyperresponsiveness (AHR) compared with the control Ova-induced mice. Histological analyses showed that RA substantially decreased the number of inflammatory cells and mucus hypersecretion in the airway. In addition, our results suggested that the protective effects of RA might be mediated by the suppression of ERK, JNK and p38 phosphorylation and activation of nuclear factor-κB (NF-κB). Furthermore, RA pretreatment resulted in a noticeable reduction in AMCase, CCL11, CCR3, Ym2 and E-selectin mRNA expression in lung tissues. These findings suggest that RA may effectively delay the progression of airway inflammation.

## 1. Introduction

Asthma is a serious health problem that affects people of all ages [1]. The incidence, morbidity and mortality of this disease have been increasing at remarkable rates over the last few decades, especially in industrialized countries [2,3]. Asthma is a chronic airway disorder characterized by airway inflammation, mucus hypersecretion and airway hyperresponsiveness (AHR) [4]. Moreover, it is closely related to increased production of various inflammatory mediators, cytokines and adhesion molecules [5,6]. The Th2 cytokines IL-4, IL-5 and IL-13, which are secreted from Th2 cells, have been demonstrated to play important roles in allergic airway inflammation [6,7,8]. Airway eosinophilia and Th2 cytokines may ultimately contribute to AHR in asthma [4]. In addition, expression of acidic mammalian chitinase (AMCase) has been shown to be increased in human asthma patients and in an aeroallergen asthma mouse model [9]. It is well known that eosinophils that respond to a variety of CC chemokines, such as CCL11 (eotaxin), CCL24 (eotaxin-2) and CCL26 (eotaxin-3), are proinflammatory granulocytes with significant roles in several inflammatory diseases, including asthma [10,11].

The mitogen-activated protein kinase (MAPK) family includes three distinct stress-activated protein kinase pathways: p38, JNK and ERK [12]. p38 and ERK have been demonstrated to participate in the modulation of IL-5 and other cytokines [13]. In addition, JNK has been shown to be associated with IgE switching [14]. Further, nuclear factor-κB (NF-κB) plays important roles in the secretion of Th2 cytokines and accumulation of inflammatory cells in the airways of murine asthma models [15,16]. Therefore, there is increasing recognition that NF-κB and MAPK show promise as molecular targets for the treatment of asthma. Some previous studies have reported that persistent activation of NF-κB and inhibition of the MAPK signalling pathway in lung tissues have potential therapeutic value in the treatment of allergic asthma [17,18,19].

Rosmarinic acid (RA) is a phenolic compound that is an ester of caffeic acid and 3,4-dihydroxyphenyllactic acid. It is found in plants, such as species of the Boraginaceae family and the subfamily Nepetoideae of the Lamiaceae family. RA possesses numerous biological activities, including anti-viral, anti-bacterial, anti-inflammatory and anti-oxidant activities [20]. Our previous study has demonstrated that RA has certain protective effects in models of lung injury induced by lipopolysaccharide (LPS) *in vivo* at a dosage range of 5–20 mg/kg [21]. Therefore, we hypothesized that RA may also have protective effects in models of asthma. In the present study, we investigated the protective effects of RA (with RA administered prior to ovalbumin (Ova) challenge) in an Ova-induced allergic asthma mouse model and examined the anti-asthmatic mechanism of RA.

## 2. Results and Discussion

### 2.1. Effects of RA on Inflammatory Cells and Th2 Cytokines in Bronchoalveolar Lavage Fluid (BALF)

**Figure 1:**
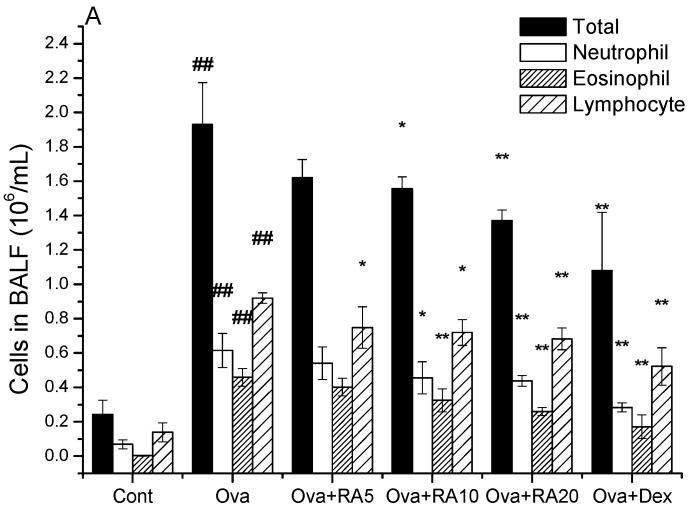

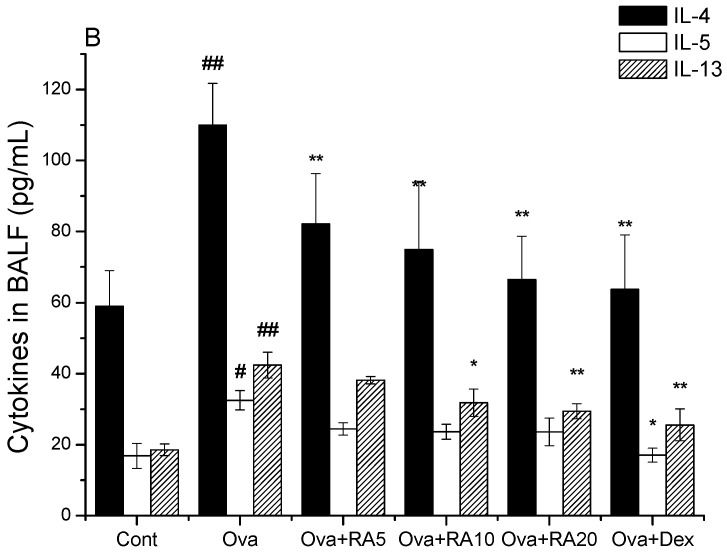
Effects of RA on inflammatory cells and secretion of Th2 cytokines in the BALF. BALF samples were obtained from the sensitized mice at 24 h after the last Ova challenge. The differential cell counts (**A**) revealed the numbers of total cells, neutrophil, eosinophil, and lymphocyte. The Th2 cytokines (**B**) were analysed via ELISA. The values represent the mean ± SEM of three independent experiments. ^#^ *p* < 0.05, ^##^ *p* < 0.01 *vs.* control group; and * *p* < 0.05, ** *p* < 0.01 *vs.* Ova.

BALF samples were collected at 24 h after the last Ova challenge. The number of inflammatory cells in the BALF was strongly increased in the Ova-treated mice compared with the phosphate-buffered serum (PBS) control mice. However, RA (20 mg/kg) or dexamethasone (Dex; 2 mg/kg) pretreatment markedly reduced the numbers of total cells, neutrophils and eosinophils (Figure 1A). In addition, the Ova-challenged mice exhibited notable increases in IL-4, IL-5 and IL-13 production in the BALF compared with the PBS control mice. However, RA (20 mg/kg) significantly inhibited IL-4 and IL-13 production compared with that in the Ova control mice (Figure 1B).

### 2.2. Effects of RA on Total IgE, Ova-Specific IgE and Eotaxin Concentrations

**Figure 2:**
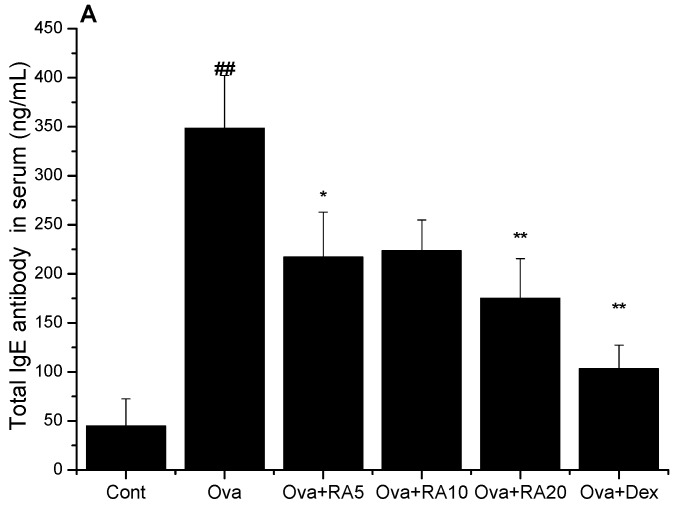

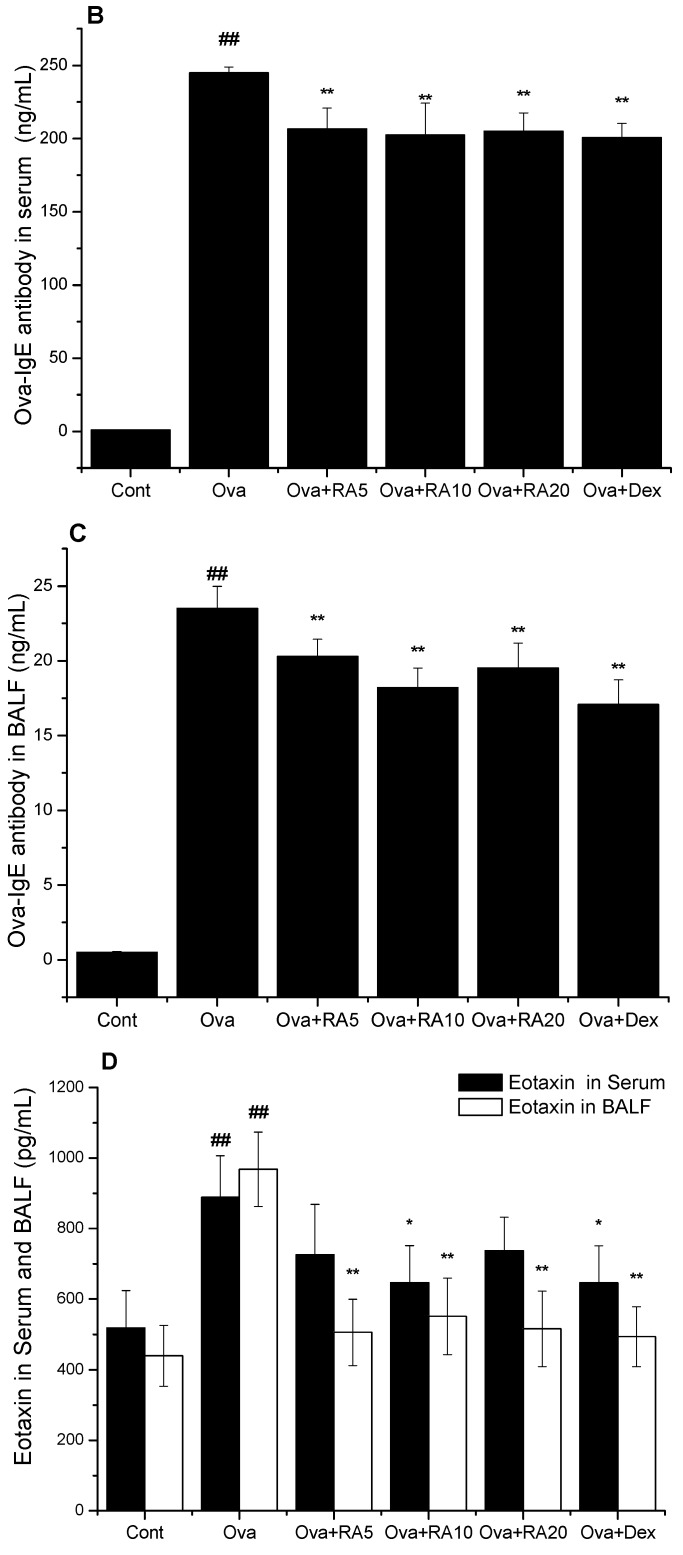
Effects of RA on the secretion of total IgE in the serum (**A**); Ova-specific IgE in the serum (**B**); Ova-specific IgE in the BALF (**C**) and eotaxin in the serum and BALF (**D**). The serum and BALF were analysed by ELISA. The values represent the mean ± SEM of three independent experiments. ^#^ *p* < 0.05, ^##^ *p* < 0.01 *vs.* control group; and * *p* < 0.05, ** *p* < 0.01 *vs.* Ova.

To determine the effects of RA on total IgE, Ova-specific IgE and eotaxin release in the Ova-challenged mice, enzyme-linked immunosorbent assay (ELISA) was performed on the serum and BALF samples. As shown in Figure 2, the total IgE, Ova-specific IgE and eotaxin concentrations in the serum and BALF were dramatically increased in the Ova-induced mice compared with the PBS control mice. However, RA (20 mg/kg) or Dex (2 mg/kg) significantly decreased the total IgE concentration in the serum. Moreover, the Ova-challenged mice pretreated with RA (5, 10 or 20 mg/kg) or Dex (2 mg/kg) exhibited marked reductions in Ova-specific IgE in the serum or BALF, as well as decreased eotaxin production in the BALF, compared with the Ova control mice.

### 2.3. Effects of RA on Histopathological Changes in Lungs

**Figure 3:**
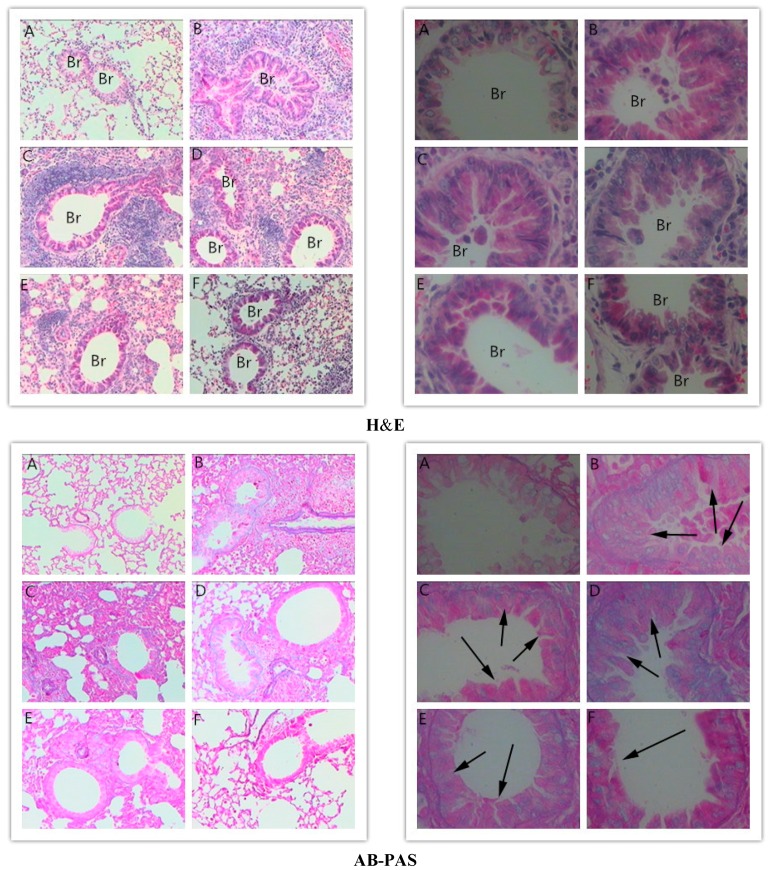
Effects of RA on airway inflammation and mucus production. Representative haematoxylin-eosin and alcian blue-periodic acid-Schiff-stained lung sections from: (**A**) PBS-challenged mice; (**B**) Ova-challenged mice; (**C**) Ova-challenged mice pretreated with RA (5 mg/kg); (**D**) Ova-challenged mice pretreated with RA (10 mg/kg); (**E**) Ova-challenged mice pretreated with RA (20 mg/kg); and (**F**) Ova-challenged mice pretreated with Dex (2 mg/kg). The left panel is magnified 100×, and the right panel is magnified 400×.

Haematoxylin and eosin (H&E) and alcian blue-periodic acid-Schiff (AB-PAS) staining were performed on the lung tissues to evaluate the effects of RA on the histological features of asthma. The lung tissues obtained from the Ova-induced mice were characterized by peribronchial inflammation due to inflammatory cell infiltration, mucus overproduction and goblet cell hyperplasia compared with those collected from the PBS-induced mice. RA (10 or 20 mg/kg) or Dex (2 mg/kg) pretreatment significantly suppressed these Ova-induced changes (Figure 3).

### 2.4. Effects of RA on AHR to Methacholine (Mch)

**Figure 4:**
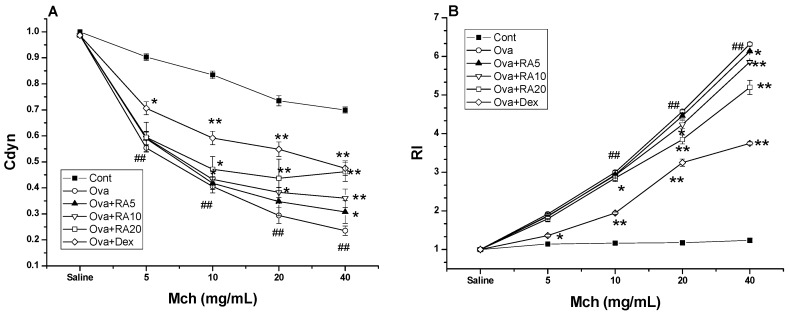
Effects of RA on Ova-induced AHR in the mice. The reduction in dynamic compliance (Cdyn) in response to methacholine (**A**) and the increase in lung resistance (RI) in response to methacholine (**B**) were assessed by the changes relative to the basal values in response to saline. ^#^ *p* < 0.05, ^##^ *p* < 0.01 *vs.* control group; and * *p* < 0.05, ** *p* < 0.01 *vs.* Ova.

AHR was estimated as a change in airway function after challenge of the mice with aerosolized Mch via the airway. It was assessed by measuring lung resistance (RI) and dynamic compliance (Cdyn) in response to increasing doses of Mch administered to mechanically ventilated mice. The Ova-induced mice developed marked AHR, typically reflected by high RI and low Cdyn values. However, the RA pretreatment ameliorated the changes in the Cdyn (Figure 4A) and RI values (Figure 4B) in response to Mch in the Ova-treated mice compared with the Ova control mice.

### 2.5. Effects of RA on MAPK and NF-κB Activation

**Figure 5:**
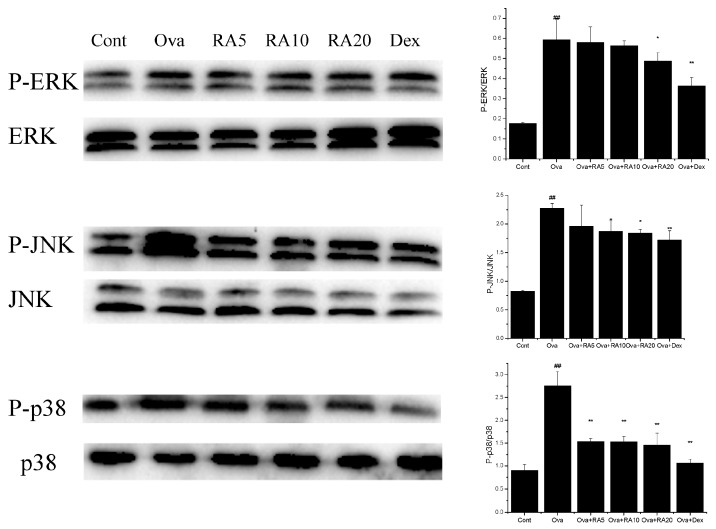
Effects of RA on the Ova-induced phosphorylation of MAPK molecules in the lungs. Cellular proteins isolated from mouse lungs were used for detection of the phosphorylated and total forms of three MAPK molecules, ERK, JNK and p38, via Western blotting. The values represent the mean ± SEM of three independent experiments. ^#^ *p* < 0.05, ^##^ *p* < 0.01 *vs.* control group; and * *p* < 0.05, ** *p* < 0.01 *vs.* Ova.

The MAPK pathway has been shown to play key roles in inflammatory responses. It also controls the synthesis and secretion of pro-inflammatory mediators secreted by activated macrophages [22]. Our data demonstrated that Ova stimulation significantly induced the phosphorylation of ERK, p38 and JNK compared with that in the PBS-induced mice. Further, RA (20 mg/kg) and Dex markedly decreased the P-ERK/ERK, P-JNK/JNK and P-p38/p38 ratios compared with those in the Ova control mice (Figure 5).

**Figure 6:**
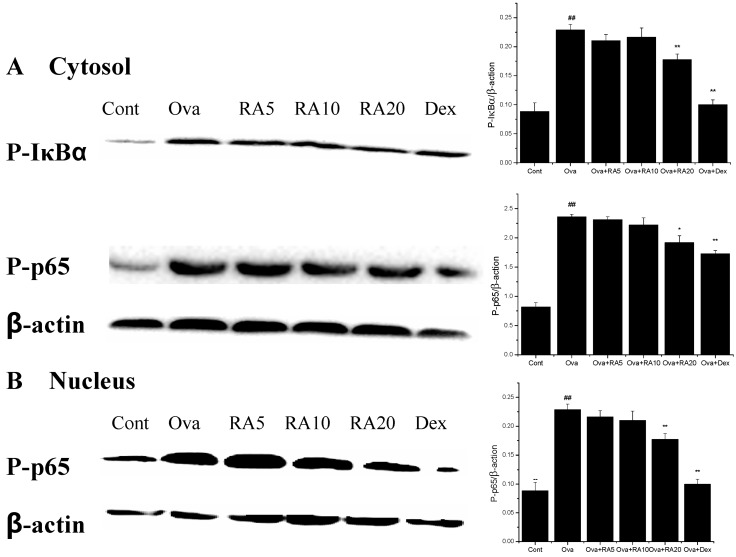
Effects of RA on Ova-induced IκBα phosphorylation and NF-κB activation in the lungs. (**A**) Detection of phosphorylated IκB and p65 in the cytosol of lungs; (**B**) detection of phosphorylated p65 in the nucleus of lungs. IκBα and NF-κB phosphorylation were measured by Western blotting. The values represent the mean ± SEM of three independent experiments. ^#^ *p* < 0.05, ^##^ *p* < 0.01 *vs.* control group; and * *p* < 0.05, ** *p* < 0.01 *vs.* Ova.

NF-κB is a transcription factor that is involved in the development of the chronic features of airway inflammation in asthma [23]. We assessed whether RA modulates the NF-κB signalling pathway. Our results showed that Ova stimulation strongly promoted IκBα phosphorylation in the cytosol and translocation of the NF-κB p65 subunit into the cytosol and nucleus. However, pretreatment with RA (20 mg/kg) significantly inhibited IκBα phosphorylation (Figure 6).

### 2.6. Effects of RA on Inflammatory Gene Expression

**Figure 7:**
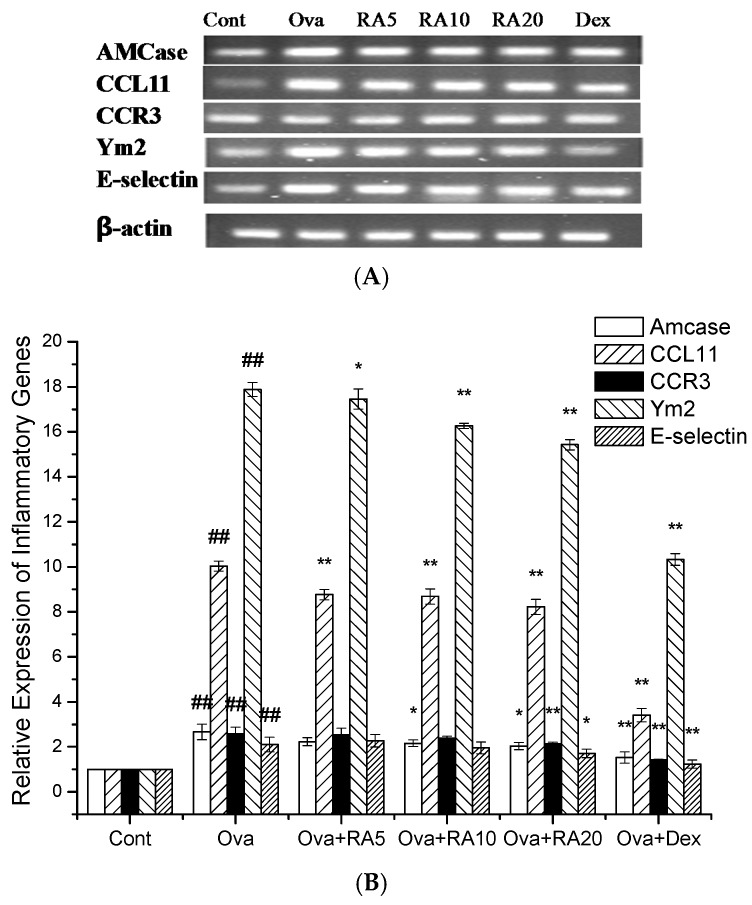
Effects of RA on Ova-induced inflammatory gene expression in the Ova-challenged mice. Lung tissues were collected at 24 h after the last Ova challenge. The PCR products were separated in a 2% agarose gel visualized under ultraviolet light (**A**). The expression levels of mRNA isolated from whole lung extracts were measured by real-time PCR (**B**). The values represent the mean ± SEM of three independent experiments. ^#^ *p* < 0.05, ^##^ *p* < 0.01 *vs.* control group; and * *p* < 0.05, ** *p* < 0.01 *vs.* Ova.

Whole lung mRNA expression was determined by quantitative RNA analysis. The chemokine CCL11 binds to a specific receptor, CCR3, which participates in eosinophil, neutrophil, and macrophage accumulation and Th2 cell recruitment [24]. The AMCase and Ym2 chitinase proteins have been demonstrated to be markedly elevated in allergic airway inflammation [9,25]. Ova challenge resulted in the dramatic up-regulation of mRNA expression of the adhesion molecule E-selectin. This increased expression is important for pulmonary infiltration of inflammatory cells [26]. Further, secretion of the chemokine CCL11 and its receptor CCR3 were markedly increased in the Ova-challenged mice compared with the PBS-induced mice, and RA pretreatment significantly reduced their secretion after Ova challenge. These results indicate that RA inhibits inflammatory cell accumulation, airway inflammation and mucus hypersecretion. Thus, we next investigated the mRNA expression of E-selectin, AMCase and Ym2 and found that RA pretreatment greatly suppressed their expression in the allergic airways (Figure 7).

### 2.7. Discussion

Natural products are used as herbal drugs for treating chronic diseases or as raw materials with particular biological activities. Thus, they are becoming increasingly significant pharmacotherapeutic sources [27]. RA has anti-viral, anti-bacterial, anti-inflammatory and anti-oxidant biological activities and is a phenolic compound present in plants, such as species of the Boraginaceae family and the subfamily Nepetoideae of the Lamiaceae family [20]. In our previous study, we have found that RA has certain anti-inflammatory effects in models of LPS-induced lung injury [21]. Therefore, we wanted to examine the protective effects of RA in a murine model of asthma. In addition, the protective effects of RA on airway inflammation in asthma are still largely unknown. This study is the first to provide experimental evidence that RA suppresses Ova-induced airway inflammation in a murine model of asthma. RA administration significantly suppressed the features of asthma by interfering with the activities of Th2 cytokines and chemokines, regulating the phosphorylation of MAPK and NF-κB and modulating the mRNA expression of inflammatory genes.

Early studies have demonstrated that allergic asthma is closely related to Th2 cytokine expression [28]. Modulation of Th2 cytokines, such as IL-4, IL-5 and IL-13, is vital for the development of typical allergic responses, including asthma. These Th2 cytokines stimulate inflammatory responses, such as inflammatory cell recruitment, mucus hypersecretion and IgE over-production [29,30,31], which are the primary pathophysiological symptoms of allergic airway diseases. Here, using an Ova-induced mouse model of asthma, our results showed that RA (5, 10 or 20 mg/kg) pretreatment suppressed the secretion of IL-4 (Figure 1), IgE and eotaxin (Figure 2). Eotaxin has been shown to play key roles in eosinophil migration and activation [32], which supports the effects of RA described herein. RA also ameliorated Ova-induced goblet cell hyperplasia, mucus hypersecretion (Figure 3) and AHR (Figure 4). Furthermore, it blocked the production of these inflammatory mediators in apparent association with modulation of the MAPK and NF-κB signalling pathways (Figure 6). These findings indicate that RA inhibits Th2-dominant inflammation and AHR in Ova-induced asthma, suggesting that it has beneficial effects on attenuating asthmatic responses.

AHR is an important feature of asthma and a pathophysiologic consequence of the effects of inflammation [33]. Airway eosinophilia and the Th2 cytokines IL-4, IL- 5 and IL-13 may ultimately cause AHR in asthma [34]. Our results demonstrated that RA inhibited AHR in the Ova-induced mice (Figure 4). This inhibition may be associated with the reduction in Th2 cytokine production and eosinophil accumulation. Therefore, it is reasonable to assume that the improvement in AHR observed in the RA-pretreated mice might have resulted from the reduction in Th2 cytokines.

It is well known that NF-κB activity is up-regulated in allergic airway inflammation, both in humans with asthma and in animal models of asthma [23,35]. Many therapeutic strategies have been developed that target the NF-κB signalling pathway, such as the use of NF-κB-specific decoy oligonucleotides [3]. NF-κB stimulates the expression of numerous pro-inflammatory genes associated with the development of allergic asthma [15,36]. Our results demonstrated that the Ova-induced phosphorylation of IκBα and NF-κB p65 was decreased by RA and Dex (Figure 6). The findings of the present study are in accordance with those of Kumar *et al.* [37], who have demonstrated that treatment with Dex decreases NF-κB protein expression.

MAPK molecules control the synthesis and secretion of pro-inflammatory mediators during inflammatory responses [22]. Moreover, a p38 inhibitor has been demonstrated to effectively inhibit eosinophilic inflammation in Ova-challenged lungs of mice and guinea pigs [38]. Therefore, MAPK is a key mediator of allergic diseases. To elucidate the mechanisms by which RA reduces Th2 cytokines, we investigated the effects of RA on MAPK. Our results showed that RA (20 mg/kg) inhibited the phosphorylation of ERK, JNK and p38 (Figure 6). These results suggest that the inhibition of ERK, JNK and p38 may have potential anti-asthmatic effects.

The expression of many chitinase proteins, including AMCase and Ym2, has been recently demonstrated to be markedly increased in allergic airway inflammation in humans with asthma and in mouse models of asthma [9,25,39]. Our results showed that RA pretreatment markedly decreased AMCase mRNA expression in the Ova-induced mice (Figure 7). These data are consistent with our Th2 cytokine data. A previous study has shown that the coordinated actions of CC chemokines in the lungs orchestrate the development of allergic inflammation in a murine model of asthma [40]. Our results demonstrated that RA markedly down-regulated CCL11 and CCR3 mRNA expression in the lungs of the Ova-induced mice (Figure 7). These findings are consistent with those of the evaluations of Th2 cytokines and eotaxin. This down-regulation may have resulted from the major decrease in Th2 cytokine secretion in the RA-pretreated airways. E-selectin is pivotal for leukocyte transmigration into the airways [41]. In this study, we demonstrated that RA suppressed E-selectin mRNA expression in the Ova-challenged lungs (Figure 7). These findings support the function of RA in decreasing the number of inflammatory cells in BALF.

RA is an active component extracted from many traditional Chinese herbal medicines, such as *Sarcandra glabra* [42], *Perilla frutescens* [43] and *Clerodendranthus spicatus* [44], which are particularly suitable for cultivating or planting. In recent years, our research group has focused on developing technologies for examination of these RA-rich traditional Chinese herbal medicines, including technologies related to planting, extraction of the active components and quality control. These studies have established a foundation for subsequent practical applications of RA.

The RA doses used in this study were determined based on the results of our previous study [21]. Three doses of 5, 10, and 20 mg/kg were used to evaluate the protective effects in mouse models of Ova-induced asthma. The results demonstrated that RA exerted strongly protective effects in the Ova-induced mice at doses of up to 10 mg/kg. Notably, the effects of the treatment were not as strong when it was applied for less than four days. We hypothesize that the lower dose of 5 mg/kg might exert beneficial effects if the treatment duration is lengthened to one week or ten days. Our other study, in which we evaluated the therapeutic effects of RA (20 mg/kg) administered after Ova challenge for 1 h on Ova-induced mice, also demonstrated its strong protective effects in a mouse model of asthma [45]. Therefore, the effective dose of RA is lower than those of other active components that we have reported in previous studies, for example, the effective dose of emodin [46] and ginkgolide B [47] are 40 mg/kg, and that of geniposide [48] is 80 mg/kg. Our findings suggest that RA might be a promising therapeutic candidate for asthma.

## 3. Material and Methods

### 3.1. Animals

Female BALB/c mice weighing approximately 18–20 g were purchased from the Centre of Experimental Animals of Guangdong (Foshan, China). The mice were housed in micro-isolator cages for 7 days for the pre-clinical studies and received food and water *ad libitum*. The laboratory temperature was 24 ± 1 °C, with a relative humidity of 40%–80%. Experiments were approved by the Ethical Committee on Animal Research of Guangxi University (Protocol number GXU2015-008). All animal experiments were performed in accordance with the Guide for the Care and Use of Laboratory Animals published by the US National Institutes of Health.

### 3.2. Reagents

**Figure 8:**
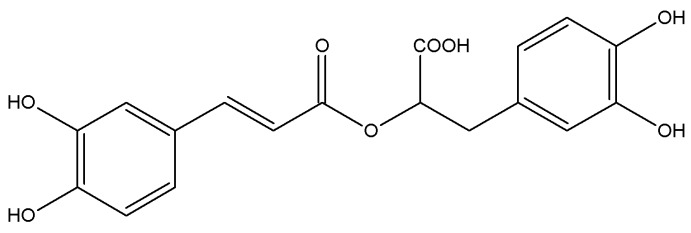
Chemical structure of RA.

IL-4, IL-5 and IL-13 ELISA kits were purchased from Neobioscience (Shenzhen, China). Total IgE and anti-Ova IgE ELISA kits were purchased from Cayman Chemical (Ann Arbor, MI, USA). A Mouse Eotaxin Platinum ELISA Kit was purchased from Affymetrix (eBioscience, Vienna, Austria). Ova (grade V) was purchased from Sigma-Aldrich (St. Louis, MO, USA). RA (purity > 98%, Figure 8) and Dex (purity: N 99.7%) were purchased from the National Institute for Food and Drug Control (Beijing, China). A MAPK Family Antibody Sampler Kit, Phospho-MAPK Family Antibody Sampler Kit and NF-κB Pathway Sampler Kit were obtained from Cell Signalling Technologies, Inc. (Beverly, MA, USA). The purities of all chemical reagents were at least analytical grade.

### 3.3. Grouping, Sensitization, Challenge and Pretreatment of Mice

**Figure 9:**
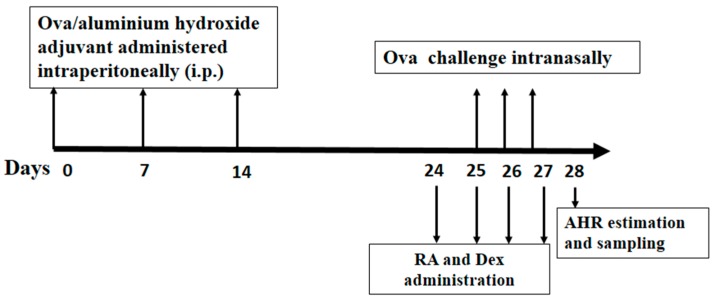
Experimental protocol for the development of allergic asthma and pretreatment with RA or Dex using a murine model. Mice were divided into six groups (*n* = 12 in each group) and sensitized using a 100 μg/mL Ova solution on days 0, 7, and 14. Subsequently, the mice were challenged with a 2 mg/mL Ova solution on days 25–27. Then, they were injected intraperitoneally with RA (5, 10 or 20 mg/kg) or Dex (2 mg/kg) on days 24–27 at 1 h prior to Ova challenge on days 25–27. Control mice were sensitized and challenged with equivalent volumes of PBS without RA or Dex.

The mice were randomly assigned to the following six groups (*n* = 12 in each group): (1) Control group; (2) Ova group; (3) Ova + RA 5 group; (4) Ova + RA 10 group; (5) Ova + RA 20 group; and (6) Ova + Dex group. They were then sensitized with Ova (20 μg) adsorbed in Imject Alum (100 μg/mL) by intraperitoneal injection on days 0, 7 and 14. On days 25–27, the mice were anesthetized and intranasally challenged with Ova (100 μg) in PBS (50 μL) [49]. Negative controls were sham sensitized and challenged with PBS following the same protocol. RA and Dex were dissolved in normal saline and administered intraperitoneally. RA (5, 10 or 20 mg/kg) or Dex (2 mg/kg) was injected intraperitoneally via four daily injections at 1 h prior to Ova challenge on days 25–27. The schematic diagram of the treatment schedule is presented in Figure 9.

### 3.4. Collection of Blood and BALF

On day 28, each mouse was sacrificed. Blood samples collected via the brachial plexus were used to measure IgE and eotaxin production, and BALF samples were processed to separate cell pellets from supernatants. Briefly, BALF was collected three times through a tracheal cannula with 0.5 mL of autoclaved, ice-cold PBS (pH = 7.2) to yield a total volume of 1.3 mL [49]. The fluid recovered from each sample was centrifuged (4 °C, 3000 rpm, 10 min) to obtain cell pellets. The supernatants were kept at −80 °C until estimation of Th2 cytokine levels. The cell pellets were resuspended in PBS for the staining and counting of the numbers of different inflammatory cells using the Wright-Giemsa staining method. At least 200 cells per slide were counted.

### 3.5. Measurements of Cytokines, Chemokines and IgE Production

The IL-4, IL-5, IL-13 and eotaxin concentrations in the BALF samples were measured using sandwich ELISA kits according to the manufacturer’s instructions. Additionally, the production of total IgE and Ova-specific IgE were determined by ELISA according to the manufacturer’s instructions.

### 3.6. Histologic Analysis

Twenty-four hours after the last inhalation exposure, the mouse lungs were fixed in 4% paraformaldehyde, paraffinized, and cut into 3-μm sections. The tissue sections were then stained with H&E to assess their general morphologies and with AB-PAS for identification of goblet cells in the epithelial tissues and mucus secretions.

### 3.7. Measurements of AHR

The mice were anesthetized at 24 h after the last Ova challenge, and tracheotomy was performed as previously described [50]. The internal jugular vein was cannulated and connected to a microsyringe for intravenous administration of Mch. Cdyn and RI in response to increasing concentrations of Mch were recorded using a whole-body plethysmograph chamber (Buxco, Sharon, CT, USA) as previously described [50]. The results were expressed as the change in AHR relative to the basal level in response to saline.

### 3.8. Western Blot Analysis of MAPK and NF-κB

Lung tissues excised at 24 h after the last Ova challenge were homogenized in liquid nitrogen and incubated in a lysis buffer containing protease and phosphatase inhibitors (Roche, Basel, Switzerland) to obtain lung proteins. A total of 60–100 μg of each protein sample was first denatured at 100 °C for 5 min in Tris-Glycine SDS Sample Loading Buffer (Beyotime, Nanjing, China) and then loaded into the lanes of an immunoblot. Next, the proteins were separated by sodium dodecyl sulphate-polyacrylamide gel electrophoresis (SDS-PAGE) and transferred to polyvinylidene difluoride (PVDF) membranes. The membranes were blocked in 5% fat-free dry milk for 2 h at room temperature. They were then incubated with primary antibodies against ERK, JNK and p38 or phospho-specific antibodies against P-ERK, P-JNK, P-p38, P-p65 and P-IκBα (CST, Beverly, MA, USA) in 5% fat-free dry milk at 4 °C overnight. Next, the membranes were washed three times for 10 min each with TBST. Then, they were probed with an horseradish peroxidase (HRP)-conjugated goat anti-rabbit (1:1000) or anti-mouse secondary antibody (1:1000) at 37 °C for 1 h. They were then washed three times with TBST and processed with ECL plus (Millipore, Billerica, MA, USA). Western blotting was also performed for β-actin (CST) as an internal protein loading control.

### 3.9. RNA Preparation and Quantitative RT-PCR

Lung tissues were harvested 24 h after the last Ova challenge. Total RNA was isolated from the mouse lungs using RNAiso Plus (Takara Bio Inc., Otsu, Japan). Reverse transcription was performed using a first-strand cDNA synthesis kit (Takara Bio Inc.). Real-time PCR was carried out on cDNA samples using a SYBR Green system (Bio-Rad, Richmond, CA, USA). The primers for the inflammatory biomarkers used are shown in Table 1. The PCR data were analysed with the sequence detection software supplied with the instrument. Melting curve analysis was performed to control for the specificity of the amplification products. The gene-specific threshold cycle (Ct) values for the respective samples were internally normalized using the average Ct value of β-actin. The ^△^Ct values for the control samples were subtracted from the ^△^Ct values for the experimental samples (^△△^Ct). The magnitude of change in the expression of each test gene was calculated as 2^−^^△△^^Ct^ [51].

### 3.10. Statistical Analysis

All values were expressed as the mean ± standard error of the mean (SEM). Differences between mean values for normally distributed data were assessed by one-way analysis of variance (ANOVA; Dunnett’s *t*-test) and Student’s *t*-test. Differences were considered significant at a *p* < 0.05.

## 4. Conclusions

In this study, we have found that RA markedly reduces the number of inflammatory cells in the BALF, inhibits IL-4, IL-5 and IL-13 secretion in the BALF, significantly decreases total IgE and Ova-specific IgE production, markedly decreases mRNA expression of AMCase, Ym2, CCL11, CCR3 and E-selectin in lung tissues, and noticeably modulates the activation of NF-κB and MAPK. These findings support our hypothesis that RA has certain protective effects against asthma at a lower dose. Although our study has demonstrated that RA may be used as an anti-asthmatic drug, further and more comprehensive studies, such as systematic pharmaceutical research studies, safety evaluations and clinical studies, are still needed before its full clinical application.

## Figures and Tables

**Table 1:** Primer sets for reverse transcription-polymerase chain reaction analysis.

Target	Forward	Reverse
Ym2	TCCACTTTGAACCACATTCCAAGGC	CGAGAGACTGAGACAGTTCAGGGA
AMCase	TGGACACACCTTCATCCTGA	CCTCAGTGGCTCCACTTCTC
CCL11	AAACCATAAACAACCTCCTC	CAATAATCCCACATCTCCTT
CCR3	TCTGCTGAGATGTCCCAATA	TCACCAACAAAGGCGTAG
E-selectin	CCCTTCCACAGAACCTACCA	TCAGCAGACATTGCTTCACC
β-actin	CTGTCCCTGTATGCCTCTG	ATGTCACGCACGATTTCC
